# Acute Cocaine Enhances Dopamine D_2_R Recognition and Signaling and Counteracts D_2_R Internalization in Sigma1R-D_2_R Heteroreceptor Complexes

**DOI:** 10.1007/s12035-019-1580-8

**Published:** 2019-04-10

**Authors:** Dasiel O. Borroto-Escuela, Manuel Narváez, Wilber Romero-Fernández, Luca Pinton, Karolina Wydra, Malgorzata Filip, Sarah Beggiato, Sergio Tanganelli, Luca Ferraro, Kjell Fuxe

**Affiliations:** 1 Department of Neuroscience, Karolinska Institutet, Biomedicum (B0851). Solnavägen 9, 171 77 Stockholm, Sweden; 2 Department of Biomolecular Science, Section of Physiology, University of Urbino, Campus Scientifico Enrico Mattei, via Ca’ le Suore 2, 610 29 Urbino, Italy; 3 Observatorio Cubano de Neurociencias, Grupo Bohío-Estudio, Zayas 50, 62100 Yaguajay, Cuba; 4 Instituto de Investigación Biomédica de Málaga, Facultad de Medicina, Universidad de Málaga, Málaga, Spain; 5 Science for Life Laboratory, Department of Cell and Molecular Biology, Uppsala University, BMC, Box 596, 751 24 Uppsala, Sweden; 6 Institute of Pharmacology, Department of Drug Addiction Pharmacology, Polish Academy of Sciences, 12 Smetna Street, 31-343 Kraków, Poland; 7 Department of Life Sciences and Biotechnology (SVEB), University of Ferrara, Ferrara, Italy

**Keywords:** Cocaine, Sigma 1 receptor, Dopamine D2 receptor, Heteroreceptor complexes, Oligomerization, Dimerization, Substance use disorder

## Abstract

The current study was performed to establish the actions of nanomolar concentrations of cocaine, not blocking the dopamine transporter, on dopamine D2 receptor (D_2_R)-sigma 1 receptor (δ1R) heteroreceptor complexes and the D_2_R protomer recognition, signaling and internalization in cellular models. We report the existence of D_2_R-δ1R heteroreceptor complexes in subcortical limbic areas as well as the dorsal striatum, with different distribution patterns using the in situ proximity ligation assay. Also, through BRET, these heteromers were demonstrated in HEK293 cells. Furthermore, saturation binding assay demonstrated that in membrane preparations of HEK293 cells coexpressing D_2_R and δ1R, cocaine (1 nM) significantly increased the D_2_R B_max_ values over cells singly expressing D_2_R. CREB reporter luc-gene assay indicated that coexpressed δ1R significantly reduced the potency of the D_2_R-like agonist quinpirole to inhibit via D_2_R activation the forskolin induced increase of the CREB signal. In contrast, the addition of 100 nM cocaine was found to markedly increase the quinpirole potency to inhibit the forskolin-induced increase of the CREB signal in the D_2_R-δ1R cells. These events were associated with a marked reduction of cocaine-induced internalization of D_2_R protomers in D_2_R-δ1R heteromer-containing cells vs D_2_R singly expressing cells as studied by means of confocal analysis of D_2_R-δ1R trafficking and internalization. Overall, the formation of D_2_R-δ1R heteromers enhanced the ability of cocaine to increase the D_2_R protomer function associated with a marked reduction of its internalization. The existence of D_2_R-δ1R heteromers opens up a new understanding of the acute actions of cocaine.

## Introduction

It recently became clear that cocaine in the nanomolar range can enhance dopamine D2 receptor (D_2_R) functions without blocking the dopamine uptake mechanism as found in neurochemical and behavioral work [1, 2]. These results indicated that cocaine can exert a direct or indirect positive allosteric modulation of the striatal D_2_R in line with the demonstration of a cocaine-induced enhancement of Gi/o coupling at striatal D_2_R [2]. Such actions are likely of relevance for the rewarding and relapse-induced effects of cocaine in view of the major role of accumbal D_2_R in mediating these actions of cocaine [3, 4]. The inhibitory D_2_R are enriched in the dorsal and ventral striato-pallidal GABA neurons, the ventral component of which is an anti-reward system [5] which becomes inhibited by D_2_R activation [6].

It is known that sigma 1 receptors (δ1R) exist in substantial densities in large numbers of central neurons including accumbal neurons [7, 8]. Cocaine was found to interact with δ1R and δ1R antagonists diminished cocaine actions [9–11]. Instead, reinforcing effects of cocaine self-administration were observed with δ1R agonists [12]. The molecular mechanism of cocaine actions in the brain can probably involve δ1R-D_1_R and δ1R-D_2_R heteromerization [13–15]. Using BRET, δ1R-D_1_R heteroreceptor complexes were demonstrated in cotransfected cells [13]. The evidence suggested a direct involvement of δ1R in mediating the cocaine-induced (150 μM) enhancement of D_1_R signaling over the Gs-AC-PKA signaling pathway.

δ1R-D_2_R heteroreceptor complexes were also demonstrated both in cotransfected cells and in striatum [14]. In cotransfected cells, cocaine in the micromolar range (30 μM) was able to partially counteract the activation by the D_2_R agonist quinpirole of the Gi/o-mediated signaling of the D_2_R. This inhibitory action of cocaine on D_2_R-mediated Gi/o signaling was mediated via the δ1R protomer [14]. These results were in sharp contrast to results obtained with 100 nM of cocaine which demonstrated that cocaine enhanced the quinpirole-induced D_2_R signaling as studied on accumbal extracellular dopamine levels and on the efficacy of dopamine to stimulate binding of GTPγS to striatal D_2_-like receptors [2]. Furthermore, the locomotor actions of quinpirole were enhanced by sub-threshold doses of cocaine. Recently, indications were obtained that D_2_R-δ1R complexes exist on striatal dopamine and glutamate nerve terminals. Nanomolar concentrations of cocaine were found to enhance the Gi/o-mediated D_2_R signaling in such complexes [16]. The possible existence of D_2_R-δ1R-N-type calcium channel heteroreceptor complexes was proposed [16].

The current study was performed to establish the actions of nanomolar concentrations of cocaine, not blocking the dopamine transporter, on δ1R-D_2L_R and δ1R-D_2S_R heteroreceptor complexes and the D_2_R protomer recognition, signaling, and internalization in cellular models. Furthermore, the distribution of the δ1R-D_2_R complexes in the ventral and dorsal striatum was evaluated using the in situ proximity ligation assay.

## Materials and Methods

### Animals

All experiments were performed using male Sprague-Dawley rats (SD) (Scanbur, Sweden). The animals were group-housed under standard laboratory conditions (20–22 °C, 50–60% humidity). Food and water available ad libitum. All studies involving animals were performed in accordance with the Stockholm North Committee on Ethics of Animal Experimentation, the Swedish National Board for Laboratory Animal and European Communities Council Directive (2010/63/EU) guidelines for accommodation and care of Laboratory Animals.

### Plasmid Constructs, Cell Culture, and Transfection

The constructs presented herein were made using standard molecular biology as described previously [17–19]. HEK293T cells were grown and transiently transfected as depicted in Borroto-Escuela et al. [20].

### Brain Tissue Samples and their Preparation

First, animals were deeply anesthetized by an intraperitoneal (i.p.) injection of a high dose of pentobarbital (60 mg/ml, [0.1 ml/100 g]) and then perfused intracardially with 30–50 mL ice-cold 4% paraformaldehyde (PFA) in 0.1 M phosphate-buffered saline (PBS, pH 7.4) solution. After perfusion, brains were collected and transferred into well-labeled glass vials filled with 4% PFA fixative solution for 6–12 h. Then, the brains were placed in 10% and 30% sucrose (0.1 M PBS, pH 7.4) and incubated for 1 day (10% sucrose) and a number of days (30% sucrose) at 4 °C with several sucrose buffer changes, until freezing the brain. The brains were frozen with isopentane and then sectioned (10–30 μm-thick) using a cryostat. The brain’s slices were stored at − 20 °C on Hoffman solution.

### In Situ Proximity Ligation Assay

To study the formation of the D_2_R-δ1R heteroreceptor complexes, the in situ proximity ligation assay (in situ PLA) was performed as described previously [20–23]. Free-floating formalin-fixed brain sections (30 μm) at Bregma level (1.0 mm) from untreated Sprague–Dawley rats were employed using the following primary antibodies: mouse monoclonal anti-D2R (MABN53, 1:600, Millipore, Sweden) and rabbit monoclonal anti-sigma1R (ab53852, 1:500, Abcam, Sweden). Control experiments employed only one primary antibody. The PLA signal was visualized and quantified by using a confocal microscope Leica TCS-SL confocal microscope (Leica, USA) and the Duolink Image Tool software. Briefly, fixed free-floating rat brain sections (storage at − 20 °C in Hoffman solution) were washed four times with PBS and quenched with 10 mM glycine buffer (0.75 g glycine in 100 ml PBS), for 20 min at room temperature. Glycine buffer is used to reduce unspecific antibodies binding and brain tissue autofluorescence. Then, after three PBS washes, incubation took place with a permeabilization buffer (10% fetal bovine serum (FBS) and 0.5% Triton X-100 or Tween 20 in Tris buffer saline (TBS), pH 7.4) for 30 min at room temperature. Again, the sections were washed twice, 5 min each, with PBS at room temperature and incubated with the blocking buffer (0.2% BSA in PBS) for 30 min at room temperature. The brain sections were then incubated with the primary antibodies diluted in a suitable concentration in the blocking solution for 1–2 h at 37 °C or at 4 °C overnight. The day after, the sections were washed twice, and the proximity probe mixture was applied to the sample and incubated for 1 h at 37 °C in a humidity chamber. The unbound proximity probes were removed by washing the slides twice, 5 min each time, with blocking solution at room temperature under gentle agitation, and the sections were incubated with the hybridization-ligation solution (BSA (250 g/ml), T4 DNA ligase (final concentration of 0.05 U/μl), Tween-20 (0.05%), NaCl 250 mM, ATP 1 mM, and the circularization or connector oligonucleotides (125–250 nM)) and incubated in a humidity chamber at 37 °C for 30 min. The excess of connector oligonucleotides was removed by washing twice, for 5 min each, with the washing buffer A (Sigma-Aldrich) Duolink Buffer A (8.8 g NaCl, 1.2 g Tris Base, 0.5 ml Tween 20 dissolved in 800 ml high purity water, pH to 7.4) at room temperature under gentle agitation, and the rolling circle amplification mixture was added to the slices and incubated in a humidity chamber for 100 min at 37 °C. Then, the sections were incubated with the detection solution in a humidity chamber at 37 °C for 30 min. In a last step, the sections were washed twice in the dark, for 10 min each, with the washing buffer B (Sigma-Aldrich, Duolink Buffer B (5.84 g NaCl, 4.24 g Tris Base, 26.0 g Tris-HCl. Dissolved in 500 ml high purity water, pH 7.5) at room temperature under gentle agitation. The free-floating sections were put on a microscope slide and a drop of appropriate mounting medium (e.g., VectaShield or Dako) was applied. The cover slip was placed on the section and sealed with nail polish. The sections were protected against light and stored for several days at − 20 °C before confocal microscope analysis.

### BRET^2^ Saturation Assay

The BRET^2^ saturation experiment was performed as described previously, see [17, 24]. Forty-eight hours after transfection, HEK293T cells, transiently transfected with constant (1 μg) or increasing amounts (0.12–5 μg) of plasmids encoding for D2R^*R*luc^ and δ1R^GFP2^, respectively, were rapidly washed twice in PBS, detached, and resuspended in the same buffer. Cell suspensions (20 μg protein) were put in duplicates into the 96-well microplate black plates with a transparent bottom (Corning 3651) (Corning, Stockholm, Sweden) for fluorescence measurement or white plates with a white bottom (Corning 3600) for BRET determination. For BRET^2^ measurements, coelenterazine-400a also called *DeepBlue*™C substrate (VWR, Sweden) was used at a final concentration of 5 μM. The readings were made 1 min after using the POLARstar Optima plate-reader (BMG Labtechnologies, Offenburg, Germany) that allows the sequential integration of the signals observed with two filter settings [410 nm (80 nm bandwidth) and 515 nm (30 nm bandwidth)]. The BRET^2^ ratio is defined as previously described by Borroto-Escuela et al. [25]. Briefly, data are represented as a normalized BRET^2^ ratio, which is defined as the BRET ratio for coexpressed Rluc and GFP2 constructs normalized against the BRET ratio found for the Rluc expression construct alone in the same experiment: BRET^2^ ratio = [(GFP^2^ emission at 515 ± 30 nm)/(Rluc emission 410 ± 80 nm)] – cf. The correction factor, cf., corresponds to (emission at 515 ± 30 nm)/(emission at 410 ± 80 nm) found with the Receptor-*R*luc construct expressed alone in the same experiment.

### BRET^2^ Competition Assay

Forty-eight hours after transfection, HEK293T cells transiently transfected with constant amounts (1 μg) of plasmids encoding for D_2L_R^*R*luc^ and δ1R^GFP2^ and increasing amounts (0.1–8 μg) of plasmids encoding for wild-type D_2L_R, D_2S_R or δ1R and the mock pcDNA3.1+, respectively. The energy transfer was determined as described for the BRET^2^ saturation assay.

### [^3^H]-Raclopride Competition Binding Experiments

Competition experiments of quinpirole (0.3 nM-3 mM) versus the D2-likeR antagonist [^3^H]-raclopride (2 nM; specific activity 78.1 Ci/mmol, PerkinElmer Life Sciences, Stockholm, Sweden) were carried out by membrane (20 μg per well) incubation at 30 °C for 90 min. Non-specific binding was defined by radioligand binding in the presence of 10 μM (+)-butaclamol (Sigma-Aldrich, Stockholm, Sweden). The incubation was terminated by rapid filtration through hydrophilic (LPB) Durapore ®Membrane (Millipore, Stockholm, Sweden) using a MultiScreen™ Vacuum Manifold 96-well (Millipore Corp, Bedford, MA), followed by five washes (200 μl per wash) with ice-cold washing buffer (50 mM Tris–HCl pH 7.4). The filters were dried, 4 ml of scintillation cocktail was added, and the bound ligand was determined after 12 h by liquid scintillation spectrometry.

### [^3^H]-Raclopride Saturation Binding Experiments

Saturation binding experiments with the D2-likeR antagonist [3H]-raclopride (specific activity 82.8 Ci/mmol, PerkinElmer Life Sciences, Sweden) were performed in membrane preparations from single and cotransfected HEK cells (100 μg protein/ml) incubated with increasing concentrations of [3H]-raclopride (ranging from 0.1 nM to 12 nM) in 250 μl of incubation buffer (50 mM Tris–HCl, 100 mM NaCl, 7 mM MgCl_2_, 1 mM EDTA, 0.05% BSA and 1 mM dithiothreitol) for 60 min at 30 °C in the presence or absence of cocaine (1 nM, 10 nM, and 100 nM) and the high affinity sigma 1 receptor antagonist PD144418 [26]. Non-specific binding was defined by radioligand binding in the presence of 10 μM (+) butaclamol (Sigma Aldrich, Sweden). The incubation was terminated by rapid filtration Whatman GF/B filters (Millipore Corp, Sweden) using a MultiScreen™ Vacuum Manifold 96-well followed by three washes (~ 250 μl per wash) with ice-cold washing buffer (50 mM Tris–HCl pH 7.4). The filters were dried, 5 ml of scintillation cocktail was added, and the bound ligand was determined after 12 h by liquid scintillation spectrometry.

### CREB Luciferase Reporter Gene Assay

A dual luciferase reporter assay has been used to indirectly detect variations of cAMP levels in transiently transfected cell lines treated with different compounds in a range of concentrations (typically 0.1 nM to 1 μM). For luciferase assays, 24 h before transfection, cells were seeded at a density of 1 × 10^6^ cells/well in 6-well dishes and transfected with Fugene (Promega, Stockholm, Sweden). Cells were co-transfected with plasmids corresponding to the four constructs as follows (per 6-well): 2 μg firefly luciferase-encoding experimental plasmid (pGL4-CRE-luc2p; Promega, Stockholm, Sweden), 1 μg of D2R plus σ1R expression vectors, and 0.5 μg Rluc-encoding internal control plasmid (phRG-B; Promega). Approximately, 46 h post-transfection, cells were incubated with appropriate ligands and harvested with passive lysis buffer (Promega, Stockholm, Sweden). The luciferase activity of cell extracts was determined using the Dual-Luciferase® Reporter (DLR™) Assay System according to the manufacturer’s protocol Promega, Stockholm, Sweden) in a POLAR star Optima plate reader (BMG Labtechnologies, Offenburg, Germany) using a 535-nm filter with a 30-nm bandwidth. Firefly luciferase was measured as firefly luciferase luminescence over a 15-s reaction period. The luciferase values were normalized against Rluc luminescence values (Luc/Rluc ratio). Chemicals used for the gene reporter assays (Raclopride, quinpirole, forskolin) were purchased from Tocris (UK) and (cocaine-HCL) Sigma Aldrich (Germany).

### Receptor Internalization Analysis by Fluorescence Confocal Microscopy

Internalization of D_2_R were evaluated by fluorescence confocal microscopy using transiently single or cotransfected HEK293T cells with constant (1 μg) amounts of plasmids encoding for Sigma1R and D_2L_R^YFP^. Cells were incubated with cocaine (100 nM) at different interval times or with a range of cocaine concentrations (1 nM–10 μM) during 30 min. Then, cells were fixed in 4% paraformaldehyde for 10 min, washed with PBS containing 20 mM glycine, and mounted in a Vectashield immunofluorescence medium (Vector Laboratories, UK). Microscope observations were performed with a × 63 oil immersion objective in a Leica TCS-SL confocal microscope (Leica, USA). The amounts of internalized D_2_R^YFP^ are shown as a single z-scan image. The rate of internalization was measured as the ratios (IntROI/membROI). The memROI is obtained by measuring the D_2_R^YFP^ fluorescence area detected at the cell surface membrane depicted by phase contrast). The IntROI is instead obtained by measuring the entire D_2_R^YFP^ fluorescence area detected in the cytoplasm of the entire cell. The basal value is obtained from cells not exposed to cocaine.

### Statistical Analysis

The number of samples (*n*) in each experimental condition is indicated in figure legends. Data from competition experiments were analyzed by nonlinear regression analysis using GraphPad Prism 5.0 (GraphPad Software Inc., San Diego, CA). The inhibition constants of the high and low affinity state of the receptor (p*K*_iH_, p*K*_iL_) from several independent replications were averaged allowing statistical comparisons using a one-way analysis of variance (ANOVA). Group differences after ANOVAs were measured by post hoc Turkey’s multiple comparison test. The *P* value 0.05 and lower was considered significant. BRET^2^ isotherms were fitted using a nonlinear regression equation assuming a single binding site, which provided BRETmax and BRET50 values.

## Results

### BRET^2^ Experiments on sigma1R and D2R Cotransfected in HEK293 Cells

**Fig. 1:**
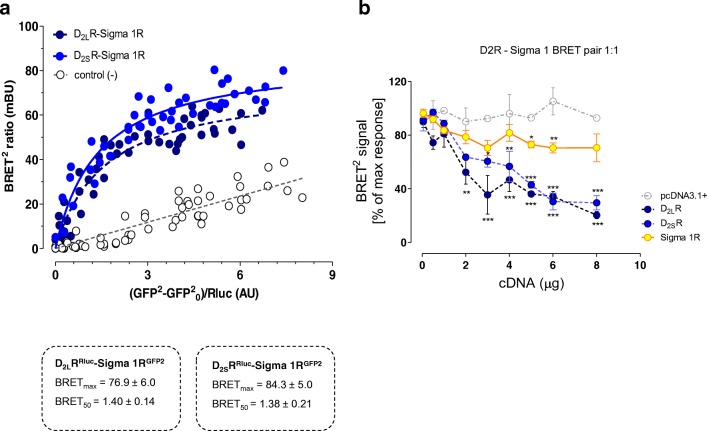
**a** BRET^2^ saturation experiments in HEK cells. Cotransfection of D_2L_^Rluc^ or D_2S_R ^Rluc^ and Sigma 1R^GFP2^ form D_2L_R^Rluc^-Sigma 1R^GFP2^ and D_2S_R^Rluc^-Sigma 1R^GFP2^ heteromers, respectively. The BRET signal obtained gives similar BRETmax and BRET 50 values, for the two heteromers demonstrated. **b** BRET^2^ competition experiments. D_2L_R and D_2S_R receptor cDNAs were shown to produce similar competition curves with marked and highly significant reductions of the BRET^2^ signal. Instead Sigma 1R cDNA in increasing concentrations only resulted in a small reduction of the BRET2 signal with a low statistical significance compared with the competition curves obtained with D_2L_R and D_2S_R cDNAs. The data represent the means ± S.E.M. of three independent experiments performed in triplicate. Statistical analysis was performed by TWO-WAY ANOVA

Saturation measurements were made with increasing amounts of δ1R^GFP2^ and a constant amount of D_2L_R and D_2S_R fused to *Renilla luciferase* (Rluc). Similar saturable BRET^2^ curves were obtained with both D_2_R isoforms (Fig. 1a). The BRETmax and BRET50 values did not differ between D_2L_R and D_2S_R. Instead, a linear relationship was found between acceptor and donor constructs in negative controls which consisted of a mixture of cells singly transfected with δ1R ^GFP2^ or D_2L_R^RLuc^ (Fig. 1a).

In competition experiments using increasing amounts of D_2L_R and D_2S_R cDNAs, similar displacement curves were observed as seen from a 70% disappearance of the BRET^2^ signal in the δ1R^GFP2^-D_2L_R^Rluc^ heteroreceptor complex using equal amounts of the two protomers (Fig. 1b). In contrast, increasing amounts of the δ1R cDNA only caused a maximal 30% disappearance of the BRET^2^ signal (Fig. 1b).

### In Situ PLA Experiments on Distribution of D_2_R-δ1R Heteroreceptor Complexes in Parts of the Rat Forebrain

**Fig. 2:**
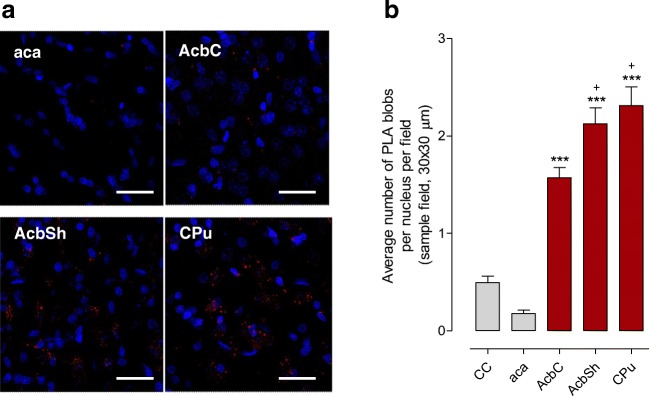
**a** PLA experiments. D_2_R-δ1R heteroreceptor complexes are seen as red clusters in nucleus accumbens core (AcbC), nucleus accumbens shell (AcbSh) and nucleus caudatus putamen (CPu), crus cerebri (cc) and anterior limb of the anterior commissure (aca). The few PLA clusters found in aca and negative controls are regarded as unspecific. Arrows point to some of the red PLA positive clusters. The length of the bar is 30 μm. **b** The density (per nucleus per sampled field) of the PLA positive complexes in AcbC, AcbSh and CPu are highly significantly different from the density found in crus cerebri and aca (***P < 0.001). The density is also significantly higher in the AcbSh and CPu versus Acb core (+*P* < 0.05). Mean ± SEM, number of rats = 5. One-way ANOVA, Tukey post-test. For sampling fields at Bregma 1.00 mm

An overall high density of PLA-positive clusters was found in the dorsal striatum (Fig. 2) based on the average number clusters per nucleus (in blue) per sample field. It was highly significantly increased vs values in negative controls and the myelinated bundles of the crus cerebri (CC) and the anterior limb of the anterior commissure (aca). In the nucleus accumbens shell, a medium density of PLA-positive D2R-δ1R heteroreceptor complexes was found and in the nucleus accumbens core a low density (Fig. 2). They were both significantly different from the number of PLA-positive clusters in cc, aca, and negative controls regarded as background values.

### Binding Experiments Using the D2 like Antagonist 3H-Raclopride in δ1R and D_2L_R Cotransfected and D_2L_R Singly Transfected HEK293 Cells

**Fig. 3:**
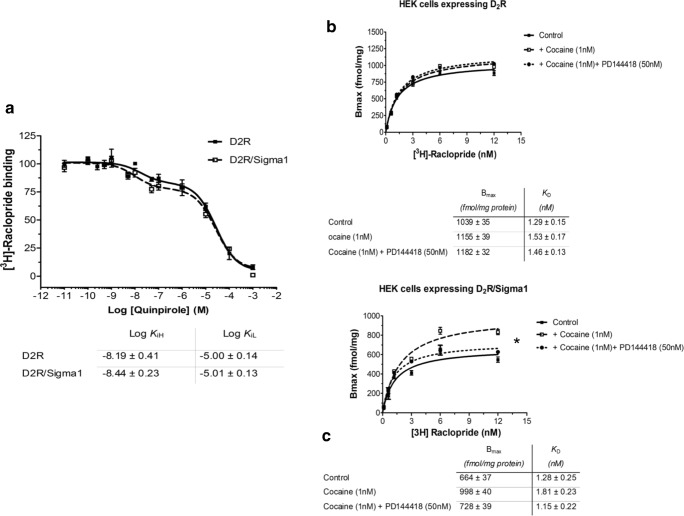
**a** 3H Raclopride competition curves with the D2likeR agonist quinpirole in D_2_R cDNA transfected HEK cells with or without cotransfection with δ1R cDNA. The KiH and KiL values obtained were similar and not significantly different. **b**, **c** In saturation binding experiments with 3H Raclopride, 1 nM of cocaine lacked effects on the Bmax and KD values in the absence of δ1R cotransfection (**b**), while a significant increase of the Bmax values (*P < 0.05) was found with cocaine (1 nM) upon cotransfection with δ1R (**c**). The action by cocaine was significantly counteracted by the sigma 1R antagonist PD144418 (50 nM) (**c**). Data are presented as the mean ± s.e.m. from five independent experiments performed in triplicate. *P < 0.05 by Student *t* test

In competition experiments, the effects of quinpirole and cocaine were examined in δ1R and D_2L_R cotransfected HEK293 cells and in singly D_2L_R transfected cells. Quinpirole produced similar competition curves in the absence or presence of δ1R (Fig. 3a). The log values for the high affinity and low affinity D2 agonist binding sites (KiH and KiL) are found below Fig. 3a. In saturation experiments, the effects of cocaine (1 nM, 10 nM, and 100 nM) were studied in cotransfected cells expressing both D_2L_R and δ1R and in singly transfected cells only expressing D_2L_R. Cocaine 1 nM produced a significant increase in the Bmax values of the 3H-Raclopride binding sites in the cotransfected cells but not in the D_2L_R singly transfected cells (Fig. 3b, c). Similar effects were also observed with cocaine 10 nM and 100 nM (data not shown). The Sigma-1R antagonist PD144418 (50 nM) blocked the action of cocaine in the cotransfected cells on the Bmax values. The K_D_ values were not significantly altered by cocaine (1 nM) neither in the absence or presence of δ1R (Fig. 3b, c).

### Signaling Experiments Using CREB Luciferase Reporter Gene Assay

**Fig. 4:**
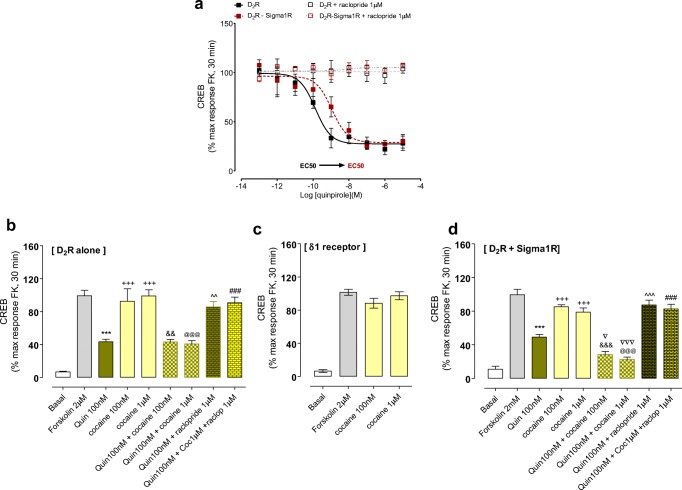
CREB signaling experiments. **a** In HEK cells cotransfected with D_2L_R and δ1R cDNAs vs cells singly transfected with D_2L_R cDNA, the inhibition of CREB signaling obtained with increasing concentrations of quinpirole was shifted to the right upon cotransfection (D_2_R (LogIC50 = −9.87 ± 0.17), D_2_R-δ1R (LogIC50 = −8.95 ± 0.22)). **b** In singly D_2_R cDNA-transfected cells the inhibition of CREB by quinpirole after forskolin-induced activation of CREB was unaffected by cocaine (100 nM and 1 μM) and cocaine alone in these concentrations lacked effects on the forskolin-induced increase in CREB signaling. The data represent the means ± S.E.M. of three independent experiments performed in triplicate. Statistical analysis was performed by one-way analysis of variance (ANOVA) followed by Tukey’s multiple comparison post-test. The *P* value 0.05 and lower was considered significant. ***Significantly different compared to Forskolin 2 μM (*P* < 0.001); +++Significantly different compared to quinpirole 100 nM (*P* < 0.001); &&Significantly different compared to cocaine 100 nM (*P* < 0.01); @@@Significantly different compared to cocaine 1 μM, (*P* < 0.001); ^^Significantly different compared to quinpirole 100 nM (*P* < 0.01); ###Significantly different compared to quinpirole 100 nM + cocaine 1 μM (*P* < 0.001). **c** In cells singly transfected cells with Sigma1R, cocaine (100 nM and 1uM) again lacked effects on the forskolin (2 μM) induced activation of CREB signaling. The data represent the means ± S.E.M. of three independent experiments performed in triplicate. Statistical analysis was performed by one-way analysis of variance (ANOVA) followed by Tukey’s multiple comparison post-test. **d** In cells cotransfected with D_2L_R and δ1R cDNAs, cocaine at 100 nM and 1 μM was instead able to significantly enhance the inhibitory actions of quinpirole on forskolin-induced increase in CREB activity. By itself, cocaine in these concentrations failed to significantly influence the forskolin-induced activation of CREB activity. The data represent the means ± S.E.M. of three independent experiments performed in triplicate. Statistical analysis was performed by one-way analysis of variance (ANOVA) followed by Tukey’s multiple comparison post-test. The *P* value 0.05 and lower was considered significant. ***Significantly different compared to Forskolin 2 μM (*P* < 0.001); +++Significantly different compared to quinpirole 100 nM (*P* < 0.001); &&&Significantly different compared to cocaine 100 nM (*P* < 0.001); @@@Significantly different compared to cocaine 1 μM (*P* < 0.001). Δ and ΔΔΔSignificantly different compared to quinpirole 100 nM (*P* < 0.05 and *P* < 0.001); ^^Significantly different compared to quinpirole 100 nM (*P* < 0.0^01); ###Significantly different compared to quinpirole 100 nM + cocaine 1 μM (*P* < 0.001). Abbreviations; raclop: raclopride, coc: cocaine, quin: quinpirole

In δ1R and D_2L_R cotransfected HEK cells quinpirole had a reduced potency to inhibit the CREB signal versus D_2L_R singly transfected cells as seen from the right shift of the competition curve (Fig. 4a).

However, the presence of cocaine (100 nM and 1 μM) enhanced the potency of the D_2_R-like agonist to inhibit the CREB signal in the cotransfected cells (Fig. 4d). Furthermore, in the cotransfected cells, cocaine alone lacked effects on the forskolin-induced increase in the CREB signal and were significantly different from the quinpirole-treated groups with or without combined treatment with cocaine (Fig. 4d).

In D_2L_R singly transfected HEK cells cocaine at 100 nM and 10 μM exerted no modulatory effects on the inhibitory actions of quinpirole to bring down the CREB signal (Fig. 4b). Furthermore, in the D_2L_R singly transfected HEK cells cocaine alone at 100 nM and 1 μM again lacked effects on the increase of the CREB signal induced by forskolin 2 μM (Fig. 4b). Nor did cocaine in these concentrations modulate the inhibitory effects of quinpirole (100 nM) on the CREB signal.

In δ1R singly transfected HEK cells cocaine alone at 100 nM and 1 μM again failed to influence the increase in the CREB signal induced by forskolin 2 μM (Fig. 4c).

### D_2L_R^YFP^ Trafficking Experiments in δ1R and D_2L_R Cotransfected and D_2L_R Singly Transfected HEK293 Cells

**Fig. 5:**
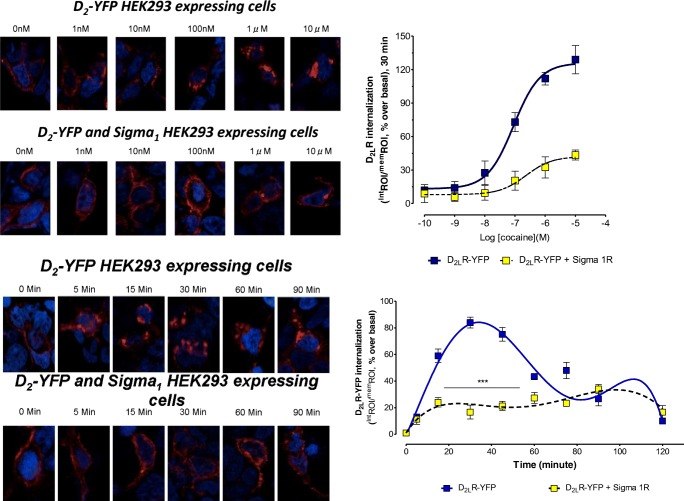
Effects of cocaine on internalization of D_2_R^YFP^ in HEK cells in the absence or presence of coexpression of Sigma1R. In the upper part, a cocaine concentration response curve on D_2_R^YFP^ internalization is illustrated in red. To the right, quantitation of the D_2_R^YFP^ internalization is found. With increasing concentrations, cocaine produces marked internalization of D_2_R^YFP^ which is counteracted by expression of Sigma1R (D_2_R (LogEC50 = −7.04 ± 0.09), D_2_R-δ1R (LogEC50 = −6.63 ± 0.40)). In the lower part, the time-course of the action of cocaine is found in the D_2_R cDNA-transfected cells in the absence or presence of Sigma1R cDNA cotransfection. Cocaine (100 nM) induces a marked internalization of D_2_R^YFP^ construct in the first hour. This action is again counteracted by the coexpression of Sigma1R with a high significance. These results are illustrated in the left lower panels, and the quantitation is found in the right lower panel. The data represent the means ± S.E.M. of three independent experiments performed with a total of eight replicates each. Statistical analysis was performed by two-way ANOVA (***P < 0.001)

The internalization of D_2L_R^YFP^ was studied in the confocal laser microscope after incubation for 30 min with different concentrations of cocaine from 1 nM to 10 μM. The internalization of D_2L_R^YFP^ was measured by determining the ratio of the total area of D_2L_R^YFP^ fluorescence in the cytoplasm, and the total area of fluorescence in the plasma membrane taken in % of this ratio in the basal state. The images obtained with different cocaine concentrations (1 nM to 10uM) of cocaine are illustrated in Fig. 5 in which the D_2L_R^YFP^ fluorescence is found in the plasma membrane, and the cytosol at different ratios between δ1R and D_2L_R cotransfected and D_2L_R singly transfected HEK293 cells.

A highly significant reduction of the D_2L_R^YFP^ internalization was observed in the δ1R and D_2L_R cotransfected versus D_2L_R singly transfected HEK293 cells after cocaine incubation. The inhibitory effects of cocaine started already at 10 nM and were clear-cut at 100 nM (Fig. 5).

The time-course of cocaine action over 90 min was also evaluated using a cocaine concentration of 100 nM with comparisons between D_2L_R^YFP^ singly expressing cells and D_2L_R^YFP^ and δ1R coexpressing cells as illustrated (Fig. 5). The cocaine-induced reduction of D_2L_R^YFP^ internalization in the D_2L_R and Sigma 1R coexpressing cells was observed during the first 60 min which was marked and highly significant.

## Discussion

Previous work demonstrated that cocaine in the nanomolar range (10 and 100 nM) can enhance the D2-like receptor function in the brain independent of its effects on the dopamine transporter and be related to direct and/or indirect allosteric actions at the D2like receptor [1, 2]. The current results in cellular models indicate that these enhancing actions of cocaine at the D_2_R in the nanomolar range are dependent on the presence of D_2_R-δ1R heteroreceptor complexes which were previously demonstrated [14, 27, 28]. The δ1R protomer appears to mediate the enhancing actions of nanomolar concentrations of cocaine on D_2_R recognition and signaling found in the present article. The latter action involves also a clear-cut counteraction of cocaine-induced D_2_R internalization. These observations underline the existence of an indirect positive allosteric modulation of D_2_R via the δ1R. The reported cocaine-induced inhibition of D_2_R signaling over Gi/o-AC in D_2_R-δ1R heteroreceptor complexes found in high micromolar concentrations [14] could not be observed with the current low concentrations used of cocaine.

The existence of D_2L_R-δ1R heteroreceptor complexes after cotransfection of the two receptors in cell lines could be validated using BRET^2^. In addition, also D_2S_R-δ1R heteroreceptor complexes were demonstrated after cotransfections with this technique. In addition, it was found in competition experiments that increasing amounts of δ1R cDNA could only reduce the BRET^2^ signal from the D_2_R-δ1R heteroreceptor complex to a minor degree. These observations can be explained by the assumption that the δ1R can interact with several domains of the D2R and not only with the D2R-δ1R interface. Thus, the δ1R may have multiple interactions with the D_2_R regions, increasing its impact on D_2_R signaling.

The involvement of δ1Rs in the cocaine action is also supported by the observation that cocaine at 1 nM could significantly increase the Bmax values of the D_2_R antagonist binding sites only in the presence of D_2_R-δ1R heteroreceptor complexes. Furthermore, this action was blocked by the δ1R antagonist PD144418. It seems possible that this action of cocaine may be linked to the D_2_R-δ1R heteroreceptor complex and involve counteraction of cocaine-induced D_2_R protomer internalization (see below).

An allosteric D_2_R-δ1R receptor–receptor interaction also appears to exist in the control of D_2L_R protomer signaling. In the analysis of the Gi/o-AC-PKA-CREB pathway using the CREB luciferase reporter gene assay, the presence of a D_2_R-δ1R heteroreceptor complex led to a reduced potency of quinpirole to inhibit the CREB signal. However, this negative allosteric receptor–receptor interaction turned into a positive interaction upon incubation with 100 nM of cocaine and restored the potency of the D_2_R agonist to inhibit this pathway. These findings indicate that with these nanomolar concentrations of cocaine, the Gi/o-mediated inhibition of the AC-PKA-CREB pathway is enhanced by cocaine not reduced as found with high 30 μM concentrations of cocaine [14].

A remarkable new action of cocaine was discovered in the current experiments on D_2_R-δ1R heteroreceptor complexes vs D_2L_R mono-homoreceptor complexes regarding effects of cocaine in nanomolar concentrations on D_2L_R internalization. Already at 10 and 100 nM cocaine produced an increase in D_2_R internalization in singly D_2L_R transfected cells, an action which saturated around 1–10 μM. In D_2_R and δ1R cotransfected cells, this action of cocaine was markedly counteracted at concentrations tested from 10 nM to 10 μM at the 30 min time-interval with a time course of inhibition lasting around 60 min. These results strongly indicate that cocaine already at nanomolar concentrations can reduce D_2L_R protomer internalization in D_2_R-δ1R heteroreceptor complexes. Through the cocaine interaction with this receptor complex, allosteric receptor–receptor interactions are altered. This may lead to reduced D_2L_R coupling to beta-arrestin which can contribute to the markedly reduced internalization and prolongation of the D_2_R protomer signaling in the D_2_R-δ1R heteroreceptor complex at the plasma membrane.

Taken together, the current results in cellular models give evidence that the cocaine action at D_2_Rs in nanomolar concentrations is substantially altered by the D_2_R presence in D_2_R-δ1R heteroreceptor complexes. The molecular mechanisms still remain to be clarified, but cocaine actions by binding to this heteroreceptor complex may alter its allosteric receptor–receptor interactions. This may lead to the demonstrated increase in Bmax values of the D_2L_R protomer and increased D_2L_R signaling over Gi/o inhibiting the AC-PKA system which reduced CREB phosphorylation. It was associated with a markedly reduced D_2L_R protomer internalization prolonging the duration of D_2_R activation. Such acute cocaine actions in the nanomolar range at the D_2_R-δ1R heteroreceptor complexes will likely contribute to understanding the role of δ1R in acute effects of cocaine on reward and seeking.
